# Editorial: Advances in immunotherapy and combination therapy for biliary tract cancers

**DOI:** 10.3389/fonc.2023.1360649

**Published:** 2024-01-08

**Authors:** Chengpei Zhu, Simon C. Robson, Huichuan Sun, Haitao Zhao

**Affiliations:** ^1^ Department of Liver Surgery, State Key Laboratory of Complex Severe and Rare Diseases, Peking Union Medical College Hospital, Chinese Academy of Medical Sciences and Peking Union Medical College (CAMS & PUMC), Beijing, China; ^2^ Department of Medicine, Division of Gastroenterology, Beth Israel Deaconess Medical Center, Harvard Medical School, Boston, MA, United States; ^3^ Department of Liver Surgery and Transplantation, Liver Cancer Institute, Zhongshan Hospital, Fudan University, Shanghai, China

**Keywords:** biliary tract cancer, immunotherapy, immune checkpoint inhibitors, systemic therapy, chemotherapy

Biliary tract cancers (BTC) are rare and aggressive tumors often diagnosed at advanced stages, with limited options for radical surgery and a poor prognosis. Combining gemcitabine with platinum-based drugs has been the standard treatment, but recent advancements in immunotherapy, especially immune checkpoint inhibitors (ICI), offer promising alternatives. This Research Topic explores the effectiveness of immunotherapy and combination therapies for BTC, investigates the molecular mechanisms and biomarkers for identifying suitable patients, and considers strategies for integrating immunotherapy into systemic treatment.

In this Research Topic, Grant et al. reported a case of pancreatic cancer with squamous cell differentiation achieved a complete pathologic response after receiving gemcitabine and cisplatin (GC) combined with nab-paclitaxel chemotherapy, suggesting that adding standard chemotherapy to this new regimen could be a valuable first-line option for pancreatic and cholangiocarcinoma. In addition, Taghizadeh et al. published an Australian expert consensus statement on locally advanced or metastatic cholangiocarcinoma that highlights the importance of adding durvalumab, an ICI, to chemotherapy as a first-line treatment option, while emphasizing GC chemotherapy regimens in first-line treatment. The consensus also highlights the necessity of selecting specific targeted therapies based on individual genetic mutations after failure of first-line therapy. These findings highlight the evolving landscape of BTC treatment, moving towards combined therapies and personalized medicine for optimal outcomes.

Furthermore, Zhang et al. demonstrated the potential of immunotherapy conversion for initially inoperable tumors. They report a patient with unresectable intrahepatic cholangiocarcinoma (ICC) who achieved successful surgery after 6 cycles of ICI plus gemcitabine and oxaliplatin (GEMOX), highlighting the ability of this combination to shrink tumors and make them operable. Additionally, Wang et al. presented a case of primary gallbladder carcinoma with liver metastases that achieved complete remission after ICI combined with GC chemotherapy followed by surgery. Tumor analysis revealed high programmed death-ligand 1 (PD-L1) expression, suggesting this patient may have been a good candidate for immunotherapy. Additionally, the presence of macrophages and CD4+ T cells indicates a positive immune response to the treatment. These cases further support the promising role of immunotherapy in BTC treatment, particularly when combined with other therapies like chemotherapy. They also emphasize the importance of tumor characteristics like PD-L1 expression in predicting response to immunotherapy. Immunological combination chemotherapy may be a way to overcome the treatment dilemma in advanced BTC. Chemotherapy may not only up-regulate checkpoint expression and alter immune cell infiltration, it also works by disrupting strategies used by tumors to evade immune recognition. The results of several clinical trials and real-world studies suggest that the addition of targeted therapies to immune-combination chemotherapy is a promising approach for the treatment of BTC, but is also challenged by the high incidence of adverse effects (1–4). Wang et al. reported a case of progressive ICC with poor results of first-line chemotherapy, which was treated with ICI plus tyrosine kinase inhibitor combined with radiotherapy in the second line to achieve better efficacy. It is also suggested that radiotherapy can largely alter the tumour microenvironment through appropriate radiation dose and fraction, thus achieving the effect of sensitizing immunotherapy.

Finally, over the past few years, advances in precision oncology have driven the development of immuno- and targeted therapies, changing the paradigm of cancer treatment, including BTC, from a population-based perspective to individualised treatment for each patient and tumour. On this basis, enabling translational surgery for unresectable BTC, thereby further enhancing the survival of BTC patients is also a future concern (5). Although immunotherapy offers great potential for BTC, there are still a majority of patients who still fail to respond well after treatment. How to identify biomarkers (such as cell-free DNA, intestinal flora, and urinary proteins) and their intrinsic mechanisms that predict the efficacy of immunotherapy is crucial for patient selection, stratified management, and socio-economic benefits, and will be one of the main directions for future attention and research.

## Author contributions

CZ: Writing – original draft, Writing – review & editing. SR: Writing – review & editing. HS: Writing – review & editing. HZ: Writing – review & editing.
